# Endothelin-1 Regulation of Exercise-Induced Changes in Flow: Dynamic Regulation of Vascular Tone

**DOI:** 10.3389/fphar.2017.00517

**Published:** 2017-10-24

**Authors:** Robert M. Rapoport, Daphne Merkus

**Affiliations:** ^1^Department of Pharmacology and Systems Physiology, University of Cincinnati College of Medicine, Cincinnati, OH, United States; ^2^Division of Experimental Cardiology, Department of Cardiology, Thoraxcenter, Cardiovascular Research School COEUR, Erasmus University Medical School Rotterdam, Rotterdam, Netherlands

**Keywords:** endothelin-1, excercise, blood flow, nitric oxide, vasoconstriction

## Abstract

Although endothelin (ET)-1 is a highly potent vasoconstrictor with considerable efficacy in numerous vascular beds, the role of endogenous ET-1 in the regulation of vascular tone remains unclear. The perspective that ET-1 plays little role in the on-going regulation of vascular tone at least under physiologic conditions is supported by findings that potential ET-1 constriction is minimized by the release of the vasodilator and ET-1 synthesis inhibitor, nitric oxide (NO). Indeed, ET-1 release and constriction is self-limited by ET-1-induced, endothelial ET_B_ receptor-mediated release of NO. Moreover, even if the balance between ET-1 and NO were reversed as the result of lowered NO activity, as occurs in a number of pathophysiologies associated with endothelial dysfunction, the well-known resistance of ET-1 constriction to reversal (as determined with exogenous ET-1) precludes ET-1 in the dynamic, i.e., moment-to-moment, regulation of vascular tone. On the other hand, and as presently reviewed, findings of ET-1-dependent modulation of organ blood flow with exercise under physiologic conditions demonstrate the dynamic regulation of vascular tone by ET-1. We speculate that this regulation is mediated at least in part through changes in ET-1 synthesis/release caused by pulsatile flow-induced shear stress and NO.

## Introduction

There is substantial evidence that increased release of the potent vasoconstrictor, endothelin (ET)-1, underlies the sustained elevated vascular tone in numerous pathophysiologies associated with endothelial dysfunction (Félétou and Vanhoutte, 2000; De Mey and Vanhoutte, 2014). In contrast, there is considerably less support for ET-1 in the regulation of vascular tone under physiologic conditions (Félétou and Vanhoutte, 2000; De Mey and Vanhoutte, 2014) although notable amongst this support is (1) endothelial ET_B_ receptor-mediated lowering of arterial pressure, due to nitric oxide (NO) release and ET-1 clearance (de Nucci et al., 1988; Gratton et al., 1997; Lavallée et al., 2001; Bourque et al., 2011; Kelland et al., 2010), and (2) ET_A_ and ET_A/B_ receptor-mediated pressure elevation due to activation of these receptors on smooth muscle, albeit findings of lowered pressure in response to ET_A_ and ET_A/B_ receptor antagonists are variable (for reviews see Lavallée et al., 2001; Bourque et al., 2011; Rapoport, 2014a) with, as presently cited, lowered pressure in pig (Merkus et al., 2002, 2003, 2006, 2007; Houweling et al., 2006; de Beer et al., 2011b; Zhou et al., 2014b) and in one human study (Mather et al., 2002), but not in other studies in human (Cardillo et al., 1999; Martin et al., 2002; Thijssen et al., 2007; Barrett-O’Keefe et al., 2013, 2015), and neither in dog (Thorin et al., 1999; Takamura et al., 2000) and rat (Maeda et al., 2002). The apparent limited role for ET-1 regulation of vascular tone under physiological conditions has been attributed in part to possible minimization of any potential ET-1 constriction by NO, which causes both vasodilation and inhibits ET-1 synthesis (Vanhoutte, 2000; De Mey and Vanhoutte, 2014).

Moreover, even if ET-1 were to regulate arterial pressure, this effect would presumably be on a sustained rather than dynamic, i.e., moment-to-moment basis (Félétou and Vanhoutte, 2000; De Mey and Vanhoutte, 2014). This conclusion follows from the widely reported observation that the ET-1 constriction is highly resistant to reversal, as demonstrated following intravenous ET-1 bolus and ET-1 washout *ex vivo* (Yanagisawa et al., 1988; Clarke et al., 1989a,b; Neubauer et al., 1990; Félétou and Vanhoutte, 2000; Meens et al., 2010, 2011; De Mey and Vanhoutte, 2014).

In apparent contradiction to these general assessments, however, it appears that with exercise not only does ET-1 serve as a physiologic regulator of vascular tone but that the regulation is dynamic (**Tables 1**, **2**). This evidence is presently reviewed and underlying mechanisms surmised.

**Table 1:** Effect of endothelin receptor antagonists on flow in non-primate mammals.

			Flow^1^			
				
Vascular bed	Species	ETRA	Rest + ETRA vs. Rest	Exercise vs. Rest	Exercise + ETRA vs. Exercise	Reference
**Coronary**	Dog	ET_A/B_	–	↑	-^2^	Takamura et al., 2000
	Pig	ET_A_	↑	↑	↓	Merkus et al., 2002
		ET_A_	↑	↑	↓^3^	Merkus et al., 2003
		ET_A/B_	↑↑	↑	↓^4^	
		ET_A_	↑	↑	↓^3^	Merkus et al., 2005
		ET_A/B_	↑	↑	↓^4^	
		ET_A/B_	↑	↑	↓	Merkus et al., 2006
		ET_A/B_	↑	↑	↓^4^	de Beer et al., 2011b
		ET_A/B_	↑	↑	↓^4^	Zhou et al., 2014a
**Lung**	Pig	ET_A_	–	↑	–	Merkus et al., 2003
		ET_A/B_	–	↑	↑	
		ET_A_	–	↑	–	Houweling et al., 2006
		ET_A/B_	–	↑	↑	
		ET_A/B_	–	↑	↑	Merkus et al., 2007
		ET_A/B_	–	↑	↑	de Beer et al., 2010
		ET_A/B_	–	↑	↑	Zhou et al., 2014b
						
**Skeletal**	Rat					Maeda et al., 2002
Plantaris		ET_A_	–	↑	↓	
		ET_A/B_	–	↑	↓	
Soleus		ET_A_	–	↑	↓	
Tibialis		ET_A_	–	↑	↓	
		ET_A/B_	–	↑	↓	
**Splanchnic**	Rat					
Intestine		ET_A_	–	↓	↑	Maeda et al., 2002
		ET_A/B_	–	↓	↑	
		ET_A_	–	↓	↑	
		ET_A/B_	–	↓	↑	
Spleen		ET_A_	–	↓	↑	
		ET_A/B_	–	↓	↑	
Stomach		ET_A_	–	↓	↑	
		ET_A/B_	–	↓	↑	


*^1^Pressure in lungs, ^2^Increased coronary sinus O_*2*_, ^3^Waned with exercise intensity, ^4^Obliterated with exercise intensity, ↑, ↓, – = increase, decrease, no change.*

**Table 2:** Effect of endothelin receptor antagonists on resting and exercise-elevated flow in humans.

				Flow		
					
Muscle	Age^1^ (yrs)	Pathophysiology	ETRA	Rest + ETRA vs. Rest	Exercise + ETRA vs. Exercise	Reference
Forearm	20–43		ET_A_	↑↑	n.d.	Verhaar et al., 1998
			ET_A/B_	↑	n.d.	
	47		ET_A_	–	n.d.	Cardillo et al., 1999
			ET_A/B_	–	n.d.	
	51		ET_A_	–	n.d.	Cardillo et al., 2000
	55	Hypercholesterolemia	ET_A_	↑	n.d.	
		Hypercholesterolemia	ET_A/B_	–	n.d.	
	18–30		ET_A_	↑	n.d.	Spratt et al., 2001
	48		ET_A_	–	n.d.	Cardillo et al., 2002
	50	Type 2 diabetes	ET_A_	↑	n.d.	
		Type 2 diabetes	ET_A/B_	↑	n.d.	
	54		ET_A/B_	↑	↑^2^	Martin et al., 2002
	54	Hypertension	ET_A/B_	↑	↑^2^	
	50		ET_A_	↑	↑	McEniery et al., 2002
	49	Hypertension	ET_A_	↑	↑↑	
	48^3^		ET_A_	–	n.d.	Cardillo et al., 2004
		Overweight	ET_A_	–	n.d.	
		Obese	ET_A_	–	n.d.	
	50^4^	Hypertension	ET_A_	–	n.d.	
		Hypertension + Overweight	ET_A_	↑	n.d.	
		Hypertension + Obese	ET_A_	↑↑	n.d.	
	55		ET_A_	–	n.d.	Shemyakin et al., 2006
			ET_A/B_	–	n.d.	
	53	Insulin resistance	ET_A_	↑	n.d.	
		Insulin resistance	ET_A/B_	–	n.d.	
	27		ET_A_	–	n.d.	Van Guilder et al., 2007
	62		ET_A_	↑	n.d.	
	27		ET_A/B_	–	n.d.	
	62		ET_A/B_	↑	n.d.	
	55		ET_A_	–	n.d.	Weil et al., 2011
	56	Overweight	ET_A_	↑	n.d.	
	57	Obese	ET_A_	↑	n.d.	
	60		ET_A/B_	–	↑^5^	Schreuder et al., 2014
	60	Type 2 diabetes	ET_A/B_	–	↑^5^	
	54		ET_A_	–	n.d.	Dow et al., 2017
	56	Overweight/Obese	ET_A_	↑	n.d.	
Leg	33		ET_A_	–	n.d.	Mather et al., 2002
	45	Type 2 diabetes	ET_A_	↑	n.d.	
	34	Obese	ET_A_	↑	n.d.	
	34		ET_A/B_	–	n.d.	Thijssen et al., 2007
	70		ET_A/B_	↑	n.d.	
	24		ET_A_	–	↑	Barrett-O’Keefe et al., 2013, 2015
	70		ET_A_	↑	↑↑	


*^1^Mean or range, ^2^Plethysmography-induced reactive hyperemia, ^3^Mean for lean, overweight and obese, ^4^Mean for hypertensive, hypertensive + overweight and hypertensive + obese, ^5^Continuous but not incremental exercise ↑, ↓, –, n.d. = increase, decrease, no change, not determined.*

## ET Receptor Antagonist Effects on Exercise-Induced Changes in Blood Flow

The effect of ET_A_/ET_A/B_ receptor antagonists, particularly when administered systemically, on local flow should be interpreted in combination with changes in local metabolism when possible. Autoregulation will attempt to maintain blood flow constant and hence vascular diameter will vary when blood pressure changes in response to systemically administered vasodilators.

### Heart

Since systemic vasodilation is accompanied by changes in heart rate and blood pressure, the oxygen demand of the myocardium is likely to be altered. Optimal methodology for the assessment of vascular tone is by relating changes in myocardial oxygen supply to changes in myocardial oxygen demand. When this is not possible, changes in coronary blood flow and/or diameter of the large coronary vessels could potentially be used as an indicator of the vasoactive effects, particularly when ET_A_/ET_A/B_ receptor antagonists are infused intracoronarily.

#### Rest

ET_A_/ET_A/B_ receptor antagonists increased coronary blood flow or dilated the coronary vasculature, depending on the species (**Table 1**). In dog, although intracoronarily (Thorin et al., 1999) and intravenously (Takamura et al., 2000) infused ET_A_ and ET_A/B_ receptor antagonists did not increase coronary flow (**Table 1**), infusion dilated the external coronary circumflex artery (Thorin et al., 1999) and increased coronary sinus O_2_ saturation, consistent with coronary dilation (Takamura et al., 2000). ET_B_ receptor antagonism and NO synthase inhibition did not alter the diameter of the external coronary circumflex artery, although coronary flow decreased (Thorin et al., 1999).

In pig, intrapulmonary artery infused ET_A_ and ET_A/B_ receptor antagonist increased flow/dilated the coronary vascular bed (Merkus et al., 2002, 2003, 2005, 2006; de Beer et al., 2011b; Zhou et al., 2014a; **Table 1**). The magnitude of increase was slightly, but significantly greater with ET_A_ receptor antagonism as compared to ET_A/B_ receptor antagonism, suggesting the presence of underlying endothelial ET_B_ receptor vasodilation (Merkus et al., 2003; **Table 1**). Along these lines, although NO synthase and cyclooxygenase inhibition did not enhance ET_A/B_ receptor antagonist increased flow, it was speculated that ischemia due to NO synthase and cyclooxygenase inhibition resulted in release of a vasodilator, e.g., adenosine, which masked the decreased flow (Merkus et al., 2006). Indeed, the possibility that ET_A/B_ receptor antagonist inhibited NO release is supported by the lack of additivity of the elevated flows by ET_A/B_ receptor antagonist and an inhibitor of phosphodiesterase-5; the latter preventing the breakdown of the NO-mediator of vasodilation, cyclic GMP (Zhou et al., 2014a). Thus, even at rest, the capacity of the endothelium to synthesize/release ET-1 is reduced by NO. It should be noted, however, that an increase in ET-1 plasma levels in the coronary vascular bed (arteriovenous difference) was not detected following NO synthase inhibition (Merkus et al., 2006).

While an explanation is not clear with respect to the enhancement and lack of effect of ET_A_/ET_A/B_ receptor antagonism on resting flow in the coronary vasculature of the pig (Merkus et al., 2002, 2003, 2005, 2006) and dog (Thorin et al., 1999; Takamura et al., 2000), respectively, one possibility is differences in the regulation of the coronary vasculature in these species (Merkus et al., 2006, 2008). In this regard, in dog coronary vasculature, cyclooxygenase and NO synthase inhibition was without effect and caused minimal constriction, respectively (as cited in Merkus et al., 2006, 2008). In contrast, in the coronary vasculature of the pig and, notably, in human, cyclooxygenase and NO synthase inhibition caused vasoconstriction (Merkus et al., 2006, 2008).

#### Exercise

ET_A_/ET_A/B_ receptor antagonists increased coronary blood flow and/or dilated the coronary vasculature, depending on the species (**Table 1**). In dog, intravenous infused ET_A/B_ receptor antagonist increased coronary sinus O_2_ saturation (Takamura et al., 2000), consistent with coronary dilation, although elevated coronary flow was not detected (Takamura et al., 2000; **Table 1**). With ventricular pacing, which did not increase coronary flow, ET_A_ and ET_A/B_ receptor antagonists increased the diameter of the external coronary circumflex artery to a similar magnitude as observed with these antagonists at rest (Thorin et al., 1999). NO synthase inhibitor did not alter the ET_A_ and ET_A/B_ receptor antagonist-increased diameter of the external coronary circumflex artery with ventricular pacing (Thorin et al., 1999).

In pig, intrapulmonary artery infused ET_A_ and ET_A/B_ receptor antagonist increased coronary dilation, with the magnitude of increase slightly but significantly greater with ET_A_ receptor antagonist at all levels of exercise intensity (Merkus et al., 2002, 2003, 2005, 2006; de Beer et al., 2011b; Zhou et al., 2014a; **Table 1**). With increasing exercise intensity, however, the additional vasodilator effect due to ET_A_ and ET_A/B_ receptor antagonism waned and was obliterated, respectively (Merkus et al., 2003, 2005; **Table 1**). As with rest, the magnitude of increase was slightly but significantly greater with ET_A_ receptor antagonism as compared to ET_A/B_ receptor antagonism (Merkus et al., 2003; **Table 1**). Contrary to the coronary ET_A_ receptor vasoconstrictor influence that waned with increasing exercise intensity, the ET_B_ receptor mediation vasodilation was similar at all exercise intensities (Merkus et al., 2003, 2006).

Despite the underlying ET_B_ receptor mediated vasodilation, neither NO synthase inhibition nor cyclooxygenase inhibition enhanced the vasodilation by ET_A_ and ET_A/B_ receptor antagonism (Merkus et al., 2006). However, in the presence of inhibitors of NO synthase and/or cyclooxygenase, the vasodilator effect of ET_A/B_ receptor blockade was sustained with increasing exercise intensity, with combined inhibition eliciting additive effects (Merkus et al., 2006). Moreover, in the presence of NO synthase and cyclooxygenase inhibitors there was a positive correlation between ET_A/B_ receptor antagonist-increased flow and exercise intensity (Merkus et al., 2006; de Beer et al., 2011b). Thus, increased exercise intensity resulted in greater NO- and prostaglandin-mediated suppression of ET-1-mediated coronary vasoconstriction (Merkus et al., 2006).

Several findings suggest that the ability of NO synthase inhibitor to prevent the ET_A/B_ receptor antagonist obliteration of increased flow is due to reversal of NO inhibition of ET-1 synthesis. Increasing exercise intensity caused a dose-dependent decrease in ET-1 synthesis in the coronary vasculature, but not in the systemic circulation, as assessed by the conversion of big ET-1 to ET-1 (de Beer et al., 2011a). NO synthase inhibition also increased ET-1 plasma levels in the coronary vasculature (coronary arteriovenous difference; Merkus et al., 2006). Decreased ET-1 synthesis with exercise is also consistent with lowered pre-pro ET-1 mRNA expression in the aorta following 30 min (shortest duration studied) in the rat (Maeda et al., 2004). Further, the NO-mediated vasodilation was not due to decreased ET-1 constriction because contractile sensitivity to infused exogenous ET-1 remained unaltered with exercise (de Beer et al., 2011a). Also, phosphodiesterase-5 inhibition and 8-Br-cGMP did not decrease ET-1 constriction, whereas the endothelium-dependent constriction to big ET-1 was decreased in isolated coronary resistance vessels (Zhou et al., 2014a). These findings are consistent with NO synthase inhibitor-increased big ET-1, but not ET-1, constriction of isolated rat mesenteric artery (Bourque et al., 2012).

#### Conclusion and Speculation

The ability of ET_A_ and ET_A/B_ receptor antagonists to increase coronary flow at rest and with exercise demonstrates that ET-1 dynamically, i.e., on a moment-to-moment basis, limits coronary flow under both conditions (**Table 1**). Moreover, the coronary endothelium has a greater capacity to release ET-1 with increased exercise intensity, an effect that is limited by NO inhibition of ET-1 synthesis. Thus, the dynamic regulation of flow by ET-1 is modulated by a dynamic balance between increased ET-1 synthesis and subsequent ET-1 release, and increased inhibition of ET-1 synthesis by enhanced NO.

It is of interest to consider that a major influence of the balance between NO and ET-1 is pulsatile shear stress. In this regard, the coronary bed is exposed to relatively high levels of shear stress even at rest (Recchia et al., 1996). Indeed, the magnitude of basal flow in the coronary vascular bed is similar to flow in exercised skeletal muscle (Laughlin et al., 1996). With increased exercise intensity, shear stress increases, resulting in further NO release and lowering ET-1 synthesis (Merkus et al., 2003, 2006; Rapoport, 2014b). Furthermore, it should be considered that along with a major effect of shear stress to increase NO release, endothelial ET_B_ receptor activation provides a relatively smaller component of NO, in particular at higher levels of exercise intensity.

### Splanchnic: Intestine, Kidney, Spleen, and Stomach

#### Rest

Intra-aortic infused ET_A_ and ET_A/B_ receptor antagonists did not alter resting flow in intestine, kidney, spleen, and stomach (Maeda et al., 2002; **Table 1**).

#### Exercise

Exercise decreased flow in intestine, kidney, spleen, and stomach (Maeda et al., 2002; **Table 1**). The amount of exercise-lowered flow was reduced by ET_A_ and ET_A/B_ receptor antagonism (Maeda et al., 2002; **Table 1**). Similar magnitudes of attenuation of the decreased flow with exercise were achieved with ET_A_ and ET_A/B_ receptor antagonists (Maeda et al., 2002; **Table 1**).

#### Conclusion and Speculation

Consistent with ET-1 as a dynamic regulator of vascular tone, is the ET_A_ and ET_A/B_ receptor antagonist reduction of exercise-induced decreased flow to intestine, kidney, spleen, and stomach (Maeda et al., 2002; **Table 1**). Thus, ET-1 appears to regulate exercise-mediated redistribution of blood flow away from the splanchnic circulation to other organs, e.g., skeletal muscle (see below; Maeda et al., 2002). The similar magnitude of ET_A_ and ET_A/B_ receptor antagonist-mediated reduction of exercise-induced decreased flow (Maeda et al., 2002; **Table 1**) suggests that ET-1 activation of endothelial ET_B_ receptors does not modulate this reduction.

The lack of effect of ET_A_ and ET_A/B_ receptor antagonism on resting flow in splanchnic organs is consistent with the lesser magnitude of sheer stress to which these organs are subjected at rest as compared to the coronary vascular bed, in which ET_A_ and ET_A/B_ receptor antagonism increased resting flow (see section “Heart”; **Table 1**). That is, ET-1 release is increased with modest elevations in shear stress and the level of NO release may be insufficient to counter, through inhibition of ET-1 synthesis, the increased ET-1 release (Rapoport, 2014b).

### Skeletal Muscle

#### Rest

ET_A_ and ET_A/B_ receptor antagonism were largely without effect on resting skeletal muscle blood flow in healthy human volunteers, as demonstrated in the forearm and leg with intra-brachial artery (Cardillo et al., 1999, 2000, 2002, 2004; Shemyakin et al., 2006; Van Guilder et al., 2007; Weil et al., 2011; Schreuder et al., 2014; Dow et al., 2017) and intra-femoral artery infused antagonist, respectively (Thijssen et al., 2007; Barrett-O’Keefe et al., 2013, 2015; **Table 2**). In contrast, other studies demonstrated increased forearm flow with intra-brachial ET_A_ and ET_A/B_ receptor antagonist infusion (Verhaar et al., 1998; Spratt et al., 2001; Martin et al., 2002; McEniery et al., 2002; **Table 2**).

An explanation for the contrasting effects of ET_A_ and ET_A/B_ receptor antagonists on resting flow does not appear to be the age of the volunteers. Specifically, while ET_A_/ET_A/B_ receptor antagonist-increased flow in forearm and leg was restricted to aged humans (Van Guilder et al., 2007; Barrett-O’Keefe et al., 2013, 2015; **Table 2**), others demonstrated increased forearm flow with ET_A_/ET_A/B_ receptor antagonism in relatively younger volunteers (Verhaar et al., 1998; Spratt et al., 2001). Another possibility is that undetected endothelial dysregulation is present in otherwise healthy volunteers. Along these lines, ET_A_/ET_A/B_ receptor antagonism increased forearm resting flow in volunteers with hypercholesterolemia, insulin resistance, type 2 diabetes, excessive weight, and combined excessive weight and hypertension, but not in normal control volunteers (Cardillo et al., 2000, 2002, 2004; Mather et al., 2002; Shemyakin et al., 2006; Weil et al., 2011; Dow et al., 2017; **Table 2**). Possibly in general support of this explanation is that intra-aorta infused ET_A_ and ET_A/B_ receptor antagonists did not lower blood flow in plantaris, soleus, and tibialis muscle of rats 10 weeks of age (Maeda et al., 2002; **Table 1**). It should also be noted, though, that other studies were unable to demonstrate increased forearm flow with ET_A_/ET_A/B_ receptor antagonism in volunteers with excessive weight and type 2 diabetes (Cardillo et al., 2004; Schreuder et al., 2014; **Table 2**).

In any case, the increased forearm flow at rest elicited by ET_A_ receptor antagonism was attributed to underlying endothelial ET_B_ receptor-mediated dilation because NO synthase inhibition and ET_B_ receptor antagonism reduced the ET_A_ receptor antagonist-induced increase in flow, and ET_B_ receptor antagonism alone decreased flow (Verhaar et al., 1998; **Table 2**). Along the general line of the involvement of NO-mitigation of the decreased flow due to ET-1, in volunteers with hypercholesterolemia and insulin resistance, ET_A_, but not ET_A/B_ receptor antagonism, increased flow (Cardillo et al., 2000; Shemyakin et al., 2006). Although, in volunteers with type 2 diabetes and aged volunteers, ET_A_ and ET_A/B_ receptor antagonism elicited similar increases in flow (Cardillo et al., 2002; Van Guilder et al., 2007). Thus, NO-mitigation of decreased flow due to ET-1 appears to depend on the disease state.

#### Exercise

In healthy human volunteers, exercise caused a similar magnitude of ET_A_ receptor antagonist-induced elevation of forearm blood flow as at rest (McEniery et al., 2002; **Table 2**). In the leg, in contrast to the lack of ET_A_ receptor antagonist-increased flow at rest, ET_A_ receptor antagonism increased flow with exercise (Barrett-O’Keefe et al., 2013, 2015). Hypertension, type 2 diabetes, and age were associated with ET_A_/ET_A/B_ receptor antagonist increased flow in forearm and leg with exercise and were even enhanced as compared to the ET_A_ receptor antagonism increased flow observed at rest (McEniery et al., 2002; Barrett-O’Keefe et al., 2013, 2015; Schreuder et al., 2014; **Table 2**). Although plethysmography-induced reactive hyperemia was increased by ET_A/B_ receptor antagonism to similar magnitudes in healthy and hypertensive volunteers (Martin et al., 2002; **Table 2**), it is not clear whether plethysmography-induced reactive hyperemia mimics the effects exercise.

In rat, intra-aortic infusion of ET_A_ and ET_A/B_ receptor antagonists actually resulted in a reduction in flow in tibialis and plantaris muscle with exercise as compared to rest (Maeda et al., 2002; **Table 1**). It should be considered, however, that the reduced flow was a direct effect of the decreased arterial pressure due to intra-aortic infusion of ET_A_ and ET_A/B_ receptor antagonist (Maeda et al., 2002).

The involvement of ET-1 production in exercise is equivocal. With one-legged exercise, ET-1 production increased in the non-exercising leg, whereas it was not changed in the exercising leg (Maeda et al., 1997). Conversely, ET-1 production was increased during knee exercise-extensor exercise in the exercising leg (Barrett-O’Keefe et al., 2013). An additional complexity is that NO formation, which is increased with exercise as demonstrated through NO synthase inhibition of dilation of the brachial artery with handgrip (Wray et al., 2011), would be anticipated to decrease ET-1 production increased by pulsatile shear stress (Rapoport, 2014b).

Changes in ET-1 contractile sensitivity of the skeletal muscle vasculature also occur with exercise. Indeed, the vasoconstrictor influence of infused ET-1 was progressively reduced with incremental exercise intensity in the leg (Wray et al., 2007).

#### Conclusion and Speculation

These findings (**Table 2**) are consistent with ET-1 as a dynamic regulator of vascular tone in the skeletal muscle with exercise. The changes in production and sensitivity together result in an altered contribution of ET-1 to skeletal muscle blood flow during exercise. The variable effects of ET-1 involvement at rest may point to different degrees of underlying endothelial dysregulation. Indeed, pathophysiologies associated with endothelial dysregulation also demonstrate dynamic ET-1 dependency of flow with rest and exercise.

### Lung

#### Rest

In lying and standing young pig (2–3 months), an intra-pulmonary artery infused ET_A_ receptor antagonist was without effect on pulmonary arterial pressure (Merkus et al., 2003, 2007; Houweling et al., 2006; de Beer et al., 2010; Zhou et al., 2014b; **Table 1**). While ET_A/B_ receptor antagonism also did not lower pulmonary arterial pressure in lying pig, in a number of studies pressure was lowered in standing pig (Merkus et al., 2003, 2007; Houweling et al., 2006; de Beer et al., 2010; Zhou et al., 2014b; **Table 1**). The lack of role of ET-1 in basal tone in lying pig may be due to ongoing NO inhibition of ET-1 synthesis because NO synthase inhibition increased pulmonary vascular resistance and an ET_A/B_ receptor antagonist prevented the increase (Merkus et al., 2007). Further, in standing pig (not investigated in lying pig), pulmonary arterial pressure was reduced by phosphodiesterase-5 inhibition and ET_A/B_ receptor blockade failed to further reduce pressure in the presence of phosphodiesterase-5 inhibition (Zhou et al., 2014b).

#### Exercise

ET_A/B_, but not ET_A_ receptor antagonism, prevented exercise-induced increased pulmonary arterial pressure (Merkus et al., 2003; **Table 1**). Thus, it was concluded that the ET-1-elevated tone during exercise was mediated by (smooth muscle) ET_B_ receptors (Merkus et al., 2003). Indeed, ET_B_ receptor-mediated, ET-1-elevated tone of the pulmonary vasculature during exercise is consistent with ET_B_ receptor mediation of constriction of pulmonary resistance vessels (MacLean et al., 1994).

Also, ongoing NO release inhibits the ET-1 increase in resistance as demonstrated by enhancement of the ET_A/B_ receptor antagonism-induced decrease in pulmonary arterial pressure with NO synthase inhibition (Merkus et al., 2007). Consistent with an underlying role for NO in inhibition of ET-1 release is lack of further increase in pulmonary arterial pressure by ET_A/B_ receptor following inhibition of phosphodiesterase-5 (Zhou et al., 2014b). Furthermore, the effect of phosphodiesterase-5 inhibition to lower pulmonary arterial pressure was due to decreased ET-1 synthesis, as demonstrated by inhibition of big ET-1 constriction in isolated pulmonary resistance vessels (Zhou et al., 2014b).

#### Conclusion and Speculation

Consistent with ET-1 as a dynamic regulator of vascular tone is the ET_A/B_ receptor antagonist reduction of exercise-induced decreased pulmonary arterial pressure by ET_A/B_ receptor antagonism (**Table 1**). The release of ET-1 in response to exercise is consistent with the ability of moderate levels of pulsatile shear stress to increase ET-1 release (Rapoport, 2014b).

The lack of effect of ET_A/B_ receptor antagonism on resting pressure (lying pig) presumably reflects in part lower ET-1 release due to lesser pulsatile shear stress, i.e., similar to that observed in splanchnic organs (see section “Splanchnic: Intestine, Kidney, Spleen, and Stomach”). Another factor involved in the lack of effect of ET_A/B_ receptor antagonism at rest is NO inhibition of ET-1 synthesis. While the relative contribution of NO released in response to endothelial ET_B_ receptor activation and shear stress is not clear, it may be reasonable to consider that the relative amount of NO release by endothelial ET_B_ receptor activation is considerably greater than in the coronary vasculature (see section “Heart”).

## Overall Conclusion and Speculation

These studies demonstrate that ET-1 dynamically regulates vascular tone with exercise. The resultant changes in blood flow redistribute flow away from metabolically less demanding organs/tissue in favor of those with greater demands (Maeda et al., 2002). By extension, ET-1 alterations of vascular tone continuously diverts blood flow to organs in response to even subtle demands, i.e., beyond what is recognized as “exercise.”

Major factors which influence the ET-1-dependent regulation of vascular tone are pulsatile shear stress and NO. This influence occurs through changes in ET-1 synthesis, rather than direct effects of shear stress and NO on ET-1 contractile potency. Thus, under conditions of moderate shear stress, which increases ET-1 release, NO release is also increased, thereby limiting the release of ET-1 in response to shear stress (Rapoport, 2014b). NO derived from ET-1-induced, endothelial ET_B_ receptor activation also acts to limit ET-1 synthesis, although in organs exposed to higher levels of shear stress, e.g., heart, the relative amount of ET_B_ receptor-derived NO as compared to shear stress-derived NO may be minimal.

In possible conflict with ET-1 serving as a dynamic regulator of vascular tone is the resistance of ET-1 constriction to reversal (see section “Introduction”). While an explanation for this apparent conflict is not entirely clear, it should be noted that the resistance of ET-1 to reversal is based upon findings with exogenous ET-1. Thus, the effects of exogenous ET-1 may not entirely mimic those due to endogenous ET-1, and notably with respect to reversibility of the constriction. At least along these general lines, despite the numerous findings of functional (constriction) cross-talk between ET_A_ and ET_B_ receptors in the normal (physiologic) vasculature as uncovered with exogenous ET-1 (Rapoport and Zuccarello, 2012), demonstrations of cross-talk with endogenously released ET-1, at least under physiologic conditions, remain to be established (to our knowledge).

Possible lack of mimicry of endogenously released ET-1 by exogenous ET-1 could also involve conditions under which endogenous ET-1 elicits constriction. In this regard, calcitonin gene-related peptide (CGRP) selectively reversed constriction and prevented re-constriction of a number of isolated vessels (but not all vessels examined) challenged with exogenous ET-1 and terminated the elevated arterial pressure to intravenous bolus ET-1 injection (Meens et al., 2010, 2011). The CGRP inhibition of ET-1vasoconstriction was attributed to dissociation of the quasi-irreversible ET-1-ET_A_ complex (Hilal-Dandan et al., 1997; Meens et al., 2010). It would be of interest to investigate whether CGRP selectively prevented/reversed the ET-1-dependent component of exercise-altered flow and CGRP receptor antagonists prevented the reversal of these changes in flow.

Finally, an additional speculative explanation for the apparent contradiction that ET-1 dynamically regulates vascular tone while the constriction to (exogenous) ET-1 is resistant to reversal relates to the dependency of the maintained ET-1 constriction upon externalization of ET_A_ receptors (Marsault et al., 1993). That is, the ET-1-ET_A_ receptor complex is rapidly internalized and the ET_A_ receptor recycled to the cell membrane, whereupon the receptor is again subject to ET-1 binding (Marsault et al., 1993). The magnitude of ET-1 constriction at any particular point in time would, therefore, reflect both reversal due to internalization of the ET-1-ET_A_ receptor complex and constriction by newly released ET-1 binding to both previously unbound and recycled ET_A_ receptors. Thus, reversible ET-1-mediated constriction could be achieved if the amount of ET-1 release were actually relatively low, although it has also been speculated that the (decreasing) fraction of internalized ET-1-ET_A_ receptor complex is still coupled to signaling (Resink et al., 1990; Chun et al., 1995).

It is widely recognized that numerous vascular pathophysiologies are associated with increased ET-1-dependent tone and, furthermore, that this tone reflects an imbalance between NO and ET-1 (see section “Introduction”). Indeed, this imbalance also represents changes in the dynamic regulation of vascular tone by ET-1, with the balance favoring ET-1 release (see section “Introduction”). In this regard, ET-1 limitation of flow with exercise is enhanced in skeletal muscle with hypertension, hypercholesterolemia, type 2 diabetes and aging (Hearon and Dinenno, 2016; **Table 2**) and in the lung in pulmonary hypertension secondary to myocardial infarction (Houweling et al., 2006; Merkus et al., 2007), while the ET-1 limitation of flow is reduced in heart with myocardial infarction (Merkus et al., 2005; de Beer et al., 2011b). While these ET-1-dependent changes in flow with exercise reflect the differential influence of numerous vasoactive factors, an understanding of the detailed mechanism whereby ET-1 release is altered with exercise will provide further insight into these vascular lesions.

## Author Contributions

RR and DM: Substantial contributions to the conception of the work; drafting the work and revising it critically for important intellectual content; final approval of the version to be published; agreement to be accountable for all aspects of the work in ensuring that questions related to the accuracy or integrity of any part of the work are appropriately investigated and resolved.

## Conflict of Interest Statement

The authors declare that the research was conducted in the absence of any commercial or financial relationships that could be construed as a potential conflict of interest.
